# Supplementary material for: Cancer cluster among small village residents near the fertilizer plant in Korea
Source: PLoS One. 2021 Feb 25;16(2):e0247661. doi: 10.1371/journal.pone.0247661 (PMC7906407; doi:10.1371/journal.pone.0247661)
Supplement: S1 Appendix — (DOCX) [file pone.0247661.s001.docx]

***Sample analysis procedural methods for polycyclic aromatic hydrocarbons (PAHs) and tobacco-specific nitrosamines (TSNAs)***

1. Polycyclic aromatic hydrocarbons (PAH) measurement

1. Solid sample

- Take the sample and standard solution out of the refrigerator and put them at room temperature
- Take 10 g of sample, add 5 g of anhydrous sodium sulfate, and mix
- Add 20 μL of 0.1 mg/L internal standard mixed solution
- Add 20 mL of methylene chloride (MC) and perform 30 minutes sonication
- After centrifugation (3 minutes at 3,000 rpm), take 10 mL of MC into a test tube
- Concentrate to 200 μL using a nitrogen concentrator
- Transfer the eluent to a vial and analyze using GC-MS/MS

1. Liquid sample

- Take the sample and standard solution out of the refrigerator and put them at room temperature
- Take 400 mL of a water sample into a 500 mL separatory funnel
- Add 20 μL of mixed internal standard (phenanthrene-d_10_, pyrene-d_10_, bifenthrin-d_5_, and benzo (a)pyrene-d_12_, 500 μg/L each)
- Add 40 g of NaCl and 20 mL of MTBE
- Shake for 20 minutes at 270 rpm with a shaker
- Transfer the MTBE layer to a test tube, add 2 g of anhydrous sodium sulfate, and mix
- Transfer the organic later to a test tube, and add 200 μL of t-butanol
- Concentrate to 20 μL using a nitrogen concentrator
- Transfer the eluent to a vial and analyze using GC-MS/MS

1. Analytical condition of GC-MS/MS

| **Analytical condition of GC-MS/MS for PAH** | |
| --- | --- |
| Parameter | Condition |
| Column | DB-5ms UI(Cross-linked 5% phenyl methyl silicon, 60 m x 0.25mm I.D. x 0.25 ㎛ film thickness) |
| Carrier gas | He at 1.2 mL/min |
| Inlet mode | Splitless |
| Injection port temperature | 310 ℃ |
| Transfer line temperature | 310 ℃ |
| Oven temperature program | Rate (℃/min) Value (℃) Hold time (min) |
|  | Initial 70 2 |
|  | Ramp1 20 240 2 |
|  | Ramp2 5 310 6.5 |
| Post run time | 0 min |

2. Tobacco-specific nitrosamines (TSNA) measurement

1. Solid sample

- Take the sample and standard solution out of the refrigerator and put them at room temperature
- Take 10 g of sample, add 3 g of anhydrous sodium sulfate, mix them, and add 20 mL of MC
- Add 50 μL of 0.1 mg/L internal standard cotinine-d_3_ and perform 30 minutes sonication
- Transfer the MC layer to a test tube, add 200 μL of mobile phase, concentrate to 200 μL using a nitrogen concentrator, and perform filtering
- Transfer the eluent to a vial and analyze using LC-MS/MS

1. Liquid sample

- Take the sample and standard solution out of the refrigerator and put them at room temperature
- Take 200 mL of sample to 500 mL separatory funnels
- Add 10 μL of 1 mg/L internal standard cotinine-d_3_
- Add 25 g of NaCl and shake to melt
- Add 5 mL of solvent MC and shake for 20 minutes with a shaker
- Transfer the MC layer to a test tube, add 200 μL of mobile phase, concentrate to 200 μL using a nitrogen concentrator, and perform filtering
- Transfer the solution to a vial and analyze using LC-MS/MS

1. Analytical condition of LC-MS/MS

| **Analytical condition of LC-MS/MS for TSNA** | |
| --- | --- |
| Parameter | Condition |
| Column | Extend C18 (2.1mm I.D., 5.0mm length, 1.8 μm particle size) |
| Moblie phase | A: 0.002% formic acid in reagent water B: Methanol |
| Column flow rate | 0.2 mL/min |
| Injection volume | 5 μL |
| Column temperature | 40 ℃ |
| Ionization mode | Positive ion electrospray |
| Capillary voltage | 3.5 kV |
| Gas temperature | 350 ℃ |
| Gas flow | 7 L/min (nitrogen) |
