# Supplementary material for: Cancer cluster among small village residents near the fertilizer plant in Korea
Source: PLoS One. 2021 Feb 25;16(2):e0247661. doi: 10.1371/journal.pone.0247661 (PMC7906407; doi:10.1371/journal.pone.0247661)
Supplement: S1 Table — SIR: Standardized incidence ratio. CI: Confidence interval. -: If there were no cases in the Jang-jeom village in the period, the calculation was impossible and the SIRs and 95% CIs are marked with “-”. (DOCX) [file pone.0247661.s002.docx]

**S1 Table. The cancer standardized incidence ratio of the Jang-jeom village according to target population and period**

|  | Sex | Target period: 2010-16 | | | | | | | | Target period: 2001-9 | | | | | | | |
| --- | --- | --- | --- | --- | --- | --- | --- | --- | --- | --- | --- | --- | --- | --- | --- | --- | --- |
|  |  | vs Republic of Korea | | vs Jeollabuk-do | | vs Iksan-si | | vs Hamra-myeon | | vs Republic of Korea | | vs Jeollabuk-do | | vs Iksan-si | | vs Hamra-myeon | |
|  |  | SIR (95% CI) | | SIR (95% CI) | | SIR (95% CI) | | SIR (95% CI) | | SIR (95% CI) | | SIR (95% CI) | | SIR (95% CI) | | SIR (95% CI) | |
| All cancers  (C00-96) | Men | 1.86 | (0.74-3.49) | 1.82 | (0.72-3.41) | 1.81 | (0.72-3.39) | 1.82 | (0.72-3.43) | 0.97 | (0.25-2.15) | 0.95 | (0.25-2.10) | 0.97 | (0.25-2.15) | 1.01 | (0.26-2.24) |
|  | Women | 2.22 | (0.80-4.34) | 2.25 | (0.81-4.41) | 2.18 | (0.78-4.27) | 2.25 | (0.81-4.41) | 1.44 | (0.37-3.20) | 1.51 | (0.39-3.34) | 1.47 | (0.38-3.27) | 1.93 | (0.50-4.29) |
|  | Total | 2.05 | (1.09-3.31) | 2.04 | (1.08-3.30) | 2.00 | (1.06-3.24) | 2.08 | (1.10-3.36) | 1.18 | (0.50-2.13) | 1.18 | (0.51-2.15) | 1.19 | (0.51-2.16) | 1.32 | (0.56-2.40) |
| All cancers  except thyroid cancer  (C00-72, 74-96) | Men | 1.91 | (0.76-3.59) | 1.86 | (0.74-3.50) | 1.85 | (0.73-3.47) | 1.89 | (0.75-3.55) | 0.98 | (0.26-2.18) | 0.96 | (0.25-2.12) | 0.98 | (0.25-2.17) | 1.02 | (0.27-2.27) |
|  | Women | 2.60 | (0.93-5.09) | 2.67 | (0.96-5.22) | 2.55 | (0.92-5.00) | 2.62 | (0.94-5.14) | 1.63 | (0.42-3.62) | 1.71 | (0.44-3.79) | 1.64 | (0.43-3.65) | 2.05 | (0.53-4.56) |
|  | Total | 2.22 | (1.18-3.59) | 2.22 | (1.18-3.59) | 2.17 | (1.15-3.51) | 2.26 | (1.20-3.66) | 1.25 | (0.53-2.26) | 1.25 | (0.53-2.27) | 1.25 | (0.53-2.27) | 1.37 | (0.58-2.48) |
| Hepatic cancer  (C22) | Men | 2.95 | (0.00-11.56) | 2.86 | (0.00-11.22) | 3.19 | (0.00-12.51) | 5.15 | (0.00-20.18) | - | | - | | - | | - | |
|  | Women | - | | - | | - | | - | | - | | - | | - | | - | |
|  | Total | 2.10 | (0.00-8.21) | 2.19 | (0.00-8.57) | 2.41 | (0.00-9.44) | 2.81 | (0.00-11.03) | - | | - | | - | | - | |
| Skin cancer except melanoma  (C44) | Men | 14.11 | (0.01-55.33) | 11.01 | (0.00-43.18) | 11.91 | (0.00-46.68) | 6.21 | (0.00-24.36) | - | | - | | - | | - | |
|  | Women | 25.41 | (4.79-62.30) | 17.59 | (3.32-43.13) | 16.72 | (3.15-40.98) | 7.93 | (1.50-19.44) | - | | - | | - | | - | |
|  | Total | 21.14 | (5.50-46.93) | 15.17 | (3.95-33.69) | 15.17 | (3.95-33.67) | 7.68 | (2.00-17.05) | - | | - | | - | | - | |
| Gallbladder  and biliary cancer  (C23-4) | Men | 16.01 | (1.51-45.88) | 13.43 | (1.27-38.50) | 11.70 | (1.10-33.53) | 10.82 | (1.02-31.02) | 8.05 | (0.00-31.55) | 7.09 | (0.00-27.78) | 6.67 | (0.00-26.14) | 4.82 | (0.00-18.89) |
|  | Women | - | | - | | - | | - | | - | | - | | - | | - | |
|  | Total | 8.07 | (0.76-23.13) | 7.01 | (0.66-20.09) | 6.26 | (0.59-17.94) | 7.65 | (0.72-21.92) | 4.08 | (0.00-15.99) | 3.84 | (0.00-15.04) | 3.72 | (0.00-14.59) | 3.64 | (0.00-14.26) |
| Colorectal cancer  (C18-20) | Men | - | | - | | - | | - | | 1.85 | (0.00-7.26) | 1.93 | (0.00-7.55) | 1.88 | (0.00-7.36) | 1.64 | (0.00-6.45) |
|  | Women | - | | - | | - | | - | | 2.61 | (0.00-10.23) | 2.82 | (0.00-11.05) | 2.60 | (0.00-10.21) | 5.14 | (0.00-20.17) |
|  | Total | - | | - | | - | | - | | 2.19 | (0.21-6.27) | 2.32 | (0.22-6.65) | 2.22 | (0.21-6.36) | 2.46 | (0.23-7.05) |
| Gastric cancer  (C16) | Men | 3.06 | (0.29-8.78) | 2.98 | (0.28-8.54) | 3.22 | (0.30-9.23) | 2.55 | (0.24-7.30) | 1.15 | (0.00-4.50) | 1.07 | (0.00-4.19) | 1.11 | (0.00-4.34) | 1.02 | (0.00-4.00) |
|  | Women | 3.06 | (0.00-11.99) | 3.02 | (0.00-11.83) | 2.98 | (0.00-11.70) | 3.37 | (0.00-13.21) | 2.40 | (0.00-9.40) | 2.30 | (0.00-9.03) | 2.33 | (0.00-9.15) | 3.39 | (0.00-13.27) |
|  | Total | 3.13 | (0.59-7.68) | 3.08 | (0.58-7.56) | 3.20 | (0.60-7.84) | 3.10 | (0.59-7.61) | 1.57 | (0.15-4.51) | 1.48 | (0.14-4.25) | 1.52 | (0.14-4.35) | 1.46 | (0.14-4.18) |
| Breast cancer  (C50) | Men | - | | - | | - | | - | | - | | - | | - | | - | |
|  | Women | 3.58 | (0.00-14.03) | 4.21 | (0.00-16.51) | 3.90 | (0.00-15.27) | 3.85 | (0.00-15.07) | - | | - | | - | | - | |
|  | Total | 3.50 | (0.00-13.71) | 4.11 | (0.00-16.13) | 3.80 | (0.00-14.88) | 4.98 | (0.00-19.53) | - | | - | | - | | - | |
| Pancreatic cancer  (C25) | Men | - | | - | | - | | - | | - | | - | | - | | - | |
|  | Women | - | | - | | - | | - | | 10.53 | (0.00-41.30) | 10.04 | (0.00-39.36) | 7.78 | (0.00-30.52) | 18.08 | (0.01-70.87) |
|  | Total | - | | - | | - | | - | | 4.77 | (0.00-18.70) | 4.44 | (0.00-17.42) | 3.74 | (0.00-14.65) | 4.88 | (0.00-19.12) |
| Lung cancer  (C33-4) | Men | 1.47 | (0.00-5.77) | 1.44 | (0.00-5.63) | 1.53 | (0.00-6.01) | 1.44 | (0.00-5.64) | 1.29 | (0.00-5.06) | 1.27 | (0.00-4.98) | 1.42 | (0.00-5.57) | 1.56 | (0.00-6.11) |
|  | Women | 3.63 | (0.00-14.24) | 3.91 | (0.00-15.31) | 3.81 | (0.00-14.96) | 1.98 | (0.00-7.77) | 3.73 | (0.00-14.62) | 3.85 | (0.00-15.08) | 4.12 | (0.00-16.14) | 7.56 | (0.00-29.65) |
|  | Total | 2.18 | (0.21-6.25) | 2.20 | (0.21-6.30) | 2.34 | (0.22-6.69) | 1.61 | (0.15-4.62) | 2.00 | (0.19-5.72) | 1.99 | (0.19-5.71) | 2.19 | (0.21-6.27) | 2.81 | (0.26-8.05) |

SIR: standardized incidence ratio. CI: confidence interval. -: if there were no cases in the Jang-jeom village in the period, the calculation was impossible and the SIRs and 95% CIs are marked with “-”.
