# Supplementary material for: Cancer cluster among small village residents near the fertilizer plant in Korea
Source: PLoS One. 2021 Feb 25;16(2):e0247661. doi: 10.1371/journal.pone.0247661 (PMC7906407; doi:10.1371/journal.pone.0247661)
Supplement: S2 Table — HR: Hazard ration. CI: Confidence interval. *: Any covariate is not adjusted. -: If there were no cases in the Jang-jeom village in the period, the calculation was impossible and the SIRs and 95% CIs are marked with “-”. (DOCX) [file pone.0247661.s003.docx]

| **S2 Table. The hazard ratio of the living in the Jang-jeom village compared to the neighborhood area by the unadjusted model** | | | | | | |
| --- | --- | --- | --- | --- | --- | --- |
|  | Minimum observation period: 7 years | | Minimum observation period: 5 years | | Minimum observation period: 3 years | |
|  | HR* (95% CI) | | HR* (95% CI) | | HR* (95% CI) | |
| All cancers (C00-96) | 2.13 | (1.23 - 3.68) | 1.94 | (1.18 - 3.19) | 1.75 | (1.11 - 2.76) |
| All cancers except thyroid cancer (C00-72, 74-96) | 2.32 | (1.34 - 4.02) | 2.09 | (1.27 - 3.43) | 1.80 | (1.13 - 2.88) |
| Hepatic cancer (C22) | 5.83 | (1.63 - 20.91) | 3.61 | (1.06 - 12.26) | 2.26 | (0.69 - 7.41) |
| Thyroid cancer (C73) | - | | - | | 0.92 | (0.12 - 6.76) |
| Skin cancer except melanoma (C44) | 17.65 | (4.74 - 65.78) | 12.58 | (3.68 - 43.00) | 12.58 | (3.68 -43.00) |
| Gallbladder and biliary cancer (C23-4) | 16.99 | (4.56 - 63.26) | 10.78 | (3.68 -31.54) | 7.92 | (2.85 -22.00) |
| Colorectal cancer (C18-20) | 0.73 | (0.10 - 5.39) | 0.67 | (0.09 - 4.89) | 0.93 | (0.23 - 3.83) |
| Gastric cancer (C16) | 3.20 | (1.12 - 9.14) | 2.22 | (0.79 - 6.21) | 1.88 | (0.68 - 5.21) |
| Breast cancer (C50) | - | | - | | 2.56 | (0.32 -20.23) |
| Pancreatic cancer (C25) | - | | 2.16 | (0.28 - 16.89) | 1.84 | (0.24 -14.15) |
| Lung cancer (C33-4) | 2.37 | (0.72 - 7.82) | 2.53 | (0.90 - 7.14) | 2.19 | (0.78 - 6.12) |
| HR: hazard ratio. CI: confidence interval. *: any covariate is not adjusted. -: if there were no cases in the Jang-jeom village in the period, the calculation was impossible and the SIRs and 95% CIs are marked with “-”. | | | | | | |
